# Supplementary material for: Systematic review and meta-analysis of developmental assets scales: A study protocol for psychometric properties
Source: PLoS One. 2024 Sep 10;19(9):e0309909. doi: 10.1371/journal.pone.0309909 (PMC11386475; doi:10.1371/journal.pone.0309909)
Supplement: S1 Table — (DOC) [file pone.0309909.s001.doc]

**S1 Table**

*PRISMA-P (Preferred Reporting Items for Systematic review and Meta-Analysis Protocols) 2015 checklist: recommended items to address in a systematic review protocol**

| Section and topic | Item No | Checklist item |
| --- | --- | --- |
| ADMINISTRATIVE INFORMATION | | |
| Title: |  |  |
| Identification | 1a | Systematic Review and Meta-Analysis of Developmental Assets Scales: A Study Protocol for Psychometric Properties |
| Update | 1b | - |
| Registration | 2 | - |
| Authors: |  |  |
| Contact | 3a | Mojtaba Habibi Asgarabad^1,2,3,4,5^, Pardis Salehi Yegaei^2^, Elizabeth Trejos-Castillo^6^, Nazanin Seyed Yaghoubi Pour^7^, & Nora Wiium^8^  ^1^ Department of Psychology, Norwegian University of Science and Technology, Trondheim, Norway  ^2^ Health Promotion Research Center, Iran University of Medical Science, Tehran, Iran  ^3^ Department of Health Psychology, School of Behavioral Sciences and Mental Health (Tehran Psychiatric Institute), Iran University of Medical Sciences, Tehran, Iran  ^4^ Positive Youth Development Lab, Human Development and Family Sciences, Texas Tech University, Texas, USA  ^5^ Center of Excellence in Cognitive Neuropsychology, Institute for Cognitive and Brain Sciences, Shahid Beheshti University, Thran, Iran  ^6^ Human Development & Family Sciences, Texas Tech University, Texas, USA  ^7^ Department of Psychology, Faculty of Education and Psychology, University of Tabriz, Tabriz, Iran  ^8^ Department of Psychosocial Science, Faculty of Psychology, University of Bergen, Bergen, Norway  Mojtaba Habibi Asgarabad [Mojtaba.h.asgarabad@ntnu.no](mailto:Mojtaba.h.asgarabad@ntnu.no)  Pardis Salehi Yegaei [pardis_salehi2012@yahoo.com](mailto:pardis_salehi2012@yahoo.com)  Elizabeth Trejos-Castillo [elizabeth.trejos@ttu.edu](mailto:elizabeth.trejos@ttu.edu)  Nazanin Seyed Yaghoubi Pour [nazanin.s.yaghoubi@gmail.com](mailto:nazanin.s.yaghoubi@gmail.com)  Nora Wiium [nora.wiium@uib.no](mailto:nora.wiium@uib.no)  Corresponding Author Address: Department of Psychology, Norwegian University of Science and Technology, 7491 Dragvoll, Trondheim, Norway |
| Contributions | 3b | MHA and PSY contributed to the eligibility criteria development, designed the extraction form, developed the search strategy, will conduct the literature search, and will provide statistical expertise. PSY and NSYP drafted the manuscript of the protocol. MHA, PSY, ETC and and NW revised the manuscript. PSY will conduct the data screening and extraction. ETC and NW contributed to the eligibility criteria development, and will provide statistical expertise. All authors provided feedbacks and approved the final text of the protocol. |
| Amendments | 4 | - |
| Support: |  |  |
| Sources | 5a | - |
| Sponsor | 5b | - |
| Role of sponsor or funder | 5c | - |
| INTRODUCTION | | |
| Rationale | 6 | Positive youth development (PYD) is a strength-based developmental perspective that put emphasis on meaningful and constructive involvement of young individuals in their communities, educational institutions, social circles, and families with the aim of empowering and enabling them to achieve their maximum capabilities [1]. PYD concentrates on boosting young people’s strengths, establishing supportive contexts, and promoting reciprocal and constructive youth ↔ context interactions rather than maladaptive tendencies or disabilities that youngsters may encounter as they grow up, such as learning disabilities, substance abuse, antisocial conduct, and psychosocial crises precipitated by puberty [2, 3].  PYD has gained ground in organizations, community-based services, and youth-serving programs providing opportunities for young people to foster their competencies [4]. Thus, an array of frameworks over the past decades has been conceptualized and designed for defining and capturing PYD.  The Search Institute in collaboration with Benson et al [5] proposed the conceptual framework of PYD through its identification of 40 developmental assets, comprising 20 individual assets and 20 assets in the contexts of home, school, and community. To assess this model, Benson, Scales [6] developed the 58-item developmental assets profile (DAP) which has two scoring types: a) the “asset category” perspective that organizes items into measures representing eight internal and external developmental asset categories (Table 1); and b) “asset context” perspective that regroup items based on how young individuals experience these assets in various ecological contexts (i.e., personal, family, school, social, and community). DAP items are scored using a scale ranging from “not at all/rarely” to “extremely/almost always.” In terms of psychometric properties, the DAP possessed invariance over time [7], as well as acceptable to good reliability and validity in both individual projects and more common group aggregates [6]. For instance, higher scores in the developmental assets were linked to adolescent achievement [8], avoidance of risky behaviors [9], and improvement of pro-social behavior, resiliency, and leadership [7, 10].  Another scale, Youth Asset Survey (YAS; [11]), was designed to assess the associations of developmental assets with risky behaviors in a prospective study on adolescents and their parents. This 37-item survey encompasses eight subscales corresponding to eight developmental assets of family communication, peer role models, general future aspirations, responsible choices, community involvement, non-parental role models, use of time on groups/sports, and use of time on religion, along with two one-item subscales of cultural respect and good health practices (exercise/nutrition). Although the construct validity and internal consistency of YAS was supported, subscales of family communication and future aspirations showed low alpha coefficients (<.68) and the number of items was limited for two additional assets of cultural respect and good health practices (exercise/nutrition) [11]. In attempt to modify this scale, Oman, Lensch [12] conducted a longitudinal cohort study and provided an improved 68-item scale, Youth Asset Survey -Revised (YAS-R). The YAS-R appraises seven additional developmental assets, namely, religiosity, school connectedness, relationship with father, relationship with mother, general self-confidence, parental monitoring, and educational aspirations for the future.  As PYD programs and frameworks grow in popularity, there is a compelling need to globally and culturally adapt appropriate and relevant measures of developmental assets that are psychometrically sound across various settings [13]. The extensive utilization of these scales necessitates the need for a systematic review to examine the caliber of their psychometric properties, describe their plausible psychometric shortcomings and strengths, and provide the best measure for researchers. Despite the availability of developmental assets scales, no research based on our information has combined data on these scales regarding their psychometric characteristics and there is a dearth of evidence on the comparison of the most employed scales. Therefore, there is a pressing need for a summary of available developmental assets scales and their psychometric robustness to serve as a guide for choosing the right tool when conducting investigations and implementing programs.  In addition, a growing body of evidence is shedding light on the high prevalence of publication bias in the context of systematic reviews [e.g., 14] suggesting that the failure to publish completed studies might pose a challenge for systematic reviews. For instance, Silagy, Middleton [15]’ study underscored the potential bias towards favoring “positive” findings in published systematic reviews. Given these observations, a predefined protocol becomes paramount that is established before the review, articulating whether the reported review outcomes align with the original study plan, and enhancing the transparency surrounding the conduct and eventual reporting of systematic review [16]. A review protocol that is subjected to peer review contributes to preventing ad hoc decisions in the review process and reducing publication bias and selective reporting [17, 18]. |
| Objectives | 7 | The proposed systematic review will be carried out to fill the existing gap and aims to exhaustively review the characteristics and psychometric properties of measures for developmental assets. In particular, our objective is to: 1) prepare a comprehensive list of the available tools developed for developmental assets, 2) summarize the important characteristics of these tools/questionnaires (e.g., number of components/items, assessment method, language, and scoring type), 4) identify the most commonly used psychometric indexes for the evaluation of these tools/measures (e.g., reliability, validity, measurement error, responsiveness, and interpretability), 5) appraise the extent to which the measurement properties of these tools/questionnaires possess the methodological quality in accordance with the COnsensus-based Standards for the selection of health Measurement INstruments (COSMIN) criteria [19], and 6) compare the quality of the measurement properties related to these tools based on the results of COSMIN (if applicable). |
| METHODS | | |
| Eligibility criteria | 8 | The four main eligibility criteria for papers containing developmental assets scales are as follows: a) being published in English, b) targeting young people aged 10–29 in line with Catalano, Skinner [21] suggestion, and c) being published after 1998 (when developmental assets were first conceptualized by Benson, Leffert [5]). Besides these primary criteria, studies must have followed at least one of the following aims: 1) reporting quantitative information on the appropriateness or acceptability of the tools for developmental assets, 2) providing at least one of the reliability indexes of these tool, 3) containing information on the validity of these tools, or 4) used these tools to evaluate risk factors (i.e., predictor variable) and/or study outcomes. Papers with different methodological designs, such as cohort studies, on-group, randomized/non-randomized controlled trials, cross-sectional, post-intervention, and case-control studies, as well as grey literature will be included if they meet eligibility criteria. Studies will meet exclusion criteria if they: 1) contain no empirical evidence (i.e., theoretical framework discussions and editorials) and 2) are literature reviews. |
| Information sources | 9 | A quick literature review based on Medical Subject Heading (MeSH) [22] will identify keywords in two domains namely, “developmental assets,” and “tools/ questionnaire” [the extended keywords are presented in syntax search in Table 2]. A preliminary search strategy will be developed with the aid of a senior librarian. A sample search strategy for PubMed is presented in Table 2 (This syntax is subject to vary based on the final search strategy). This search strategy has been adjusted according to the second version of the COSMIN initiative’s search filter [23]. Each domain will be searched individually to launch pilot searches. Following that, a comprehensive search will be conducted by combining all domains to ensure an appropriate search strategy is implemented. Subsequently, the databases Scopus, PubMed, PsycINFO, and Web of Science will be searched from the establishment of developmental assets (1998) through the 1st of April 2024 (This date is subject to vary based on the final date of coverage). To include additional studies as well as the investigation of references’ references, a broader search of scientific journals spanning comparable fields will be conducted. Furthermore, we will contact specialists in the field to obtain unpublished or under-review papers. Ultimately, we will search gray literature through the Healthcare Management Information Consortium (HMIC) and the European Association for Grey Literature Exploitation (EAGLE). |
| Search strategy | 10 | (instrumentation[sh] OR methods[sh] OR "Validation Studies" OR "psychometrics" OR psychometr*[tiab] OR "outcome assessment"[tiab] OR "outcome measure*" OR "discriminant analysis" OR reliab*[tiab] OR unreliab*[tiab] OR valid*[tiab] OR "internal consistency"[tiab] OR (cronbach*[tiab] AND (alpha[tiab] OR alphas[tiab])) OR (item[tiab] AND (correlation*[tiab] OR selection*[tiab] OR agreement OR test-retest[tiab] OR (test[tiab] AND retest[tiab]) OR (reliab*[tiab] AND (test[tiab] OR retest[tiab])) OR stability[tiab] OR interrater[tiab] OR inter-rater[tiab] OR intrarater[tiab] OR intra-rater[tiab] OR intertester[tiab] OR intratester[tiab] OR intratester[tiab] OR intra-observer[tiab] OR kappa[tiab] OR kappa's[tiab] OR kappas[tiab] AND (measure OR measures OR (intraclass[tiab] AND correlation*[tiab]) OR "factor analysis"[tiab] OR "factor analyses"[tiab] OR "factor structure"[tiab] OR "factor structures"[tiab] OR dimension*[tiab] OR subscale*[tiab] OR (multitrait[tiab] AND scaling[tiab] AND (analysis[tiab] OR analyses[tiab])) OR "item discriminant"[tiab] OR "interscale correlation*"[tiab] OR error[tiab] OR errors[tiab] OR (uncertainty[tiab] AND (measurement[tiab] OR measuring[tiab])) OR "standard error of measurement"[tiab] OR "Item response model"[tiab] OR IRT[tiab] OR Rasch[tiab] OR "Differential item functioning"[tiab] OR DIF[tiab] OR "computer adaptive testing"[tiab] OR "item bank"[tiab] OR "cross-cultural equivalence"[tiab]) |
| Study records: |  |  |
| Data management | 11a | For arranging references, titles, and abstracts of the papers and identifying duplicates, the Rayyan QCRI online software [24] will be applied. |
| Selection process | 11b | The titles and abstracts will be examined in the screening stage, and papers that are incompatible with our study’s purpose will be excluded. In the eligibility phases, the articles’ full text will be subject to review, and any studies that fail to fulfill our inclusion criteria will be excluded and those that met the inclusion criteria will be used for meta-analysis. An independent reviewer will review the manuscripts (PSY) and a senior researcher (MHA) will review the results. The details of the screening procedure are displayed in the flowchart of the PRISMA extension for systematic reviews (Fig 1). |
| Data collection process | c11c | The data extraction form will be designed in Microsoft Excel 2016 to extract the data (a sample form is presented in Table 3). In the following step, the data from three papers will be extracted to identify and modify the form’s possible flaws and deficiencies. Two expert researchers (PSY and MHA) will extract the information from chosen papers individually and in case of any ambiguity, a senior researcher will engage (NW). In cases where data in the papers are missing, we will contact the authors and ask for original data via Email. |
| Data items | 12 | The final form of extracted data will contain details on authors, the year of publication, country, language, sample size, age, gender, classification of country and family by income level (ranging from low to high income), minority group, study setting (e.g., family/home, community, or school), tool title, tool development (with objectives) or adaptation, how initial questions were generated (e.g., theory- and literature review-derived, expert panel, focus group discussion, combining previous tools), assessment method (self/proxy/teen/teacher-report questionnaire, interview, observation), scoring type (multiple-choice, Likert, etc.), number of subscales and items, reliability (i.e., internal consistency and measurement error), validity (i.e., face, content, construct, structural, cross-cultural, criterion, known-group, and longitudinal validity), responsiveness, and interpretability. |
| Outcomes and prioritization | 13 | The core outcomes of this study include offering an exhaustive and clear description of the tools accessible for developmental and discovering the possible shortcomings and strengths of these tools. The additional outcomes are: a) aiding researchers to select appropriate tools in future investigations, and b) assisting researchers in selecting appropriate tool by taking into account the utility and adaptability of the chosen instrument in their region and cultural context. |
| Risk of bias in individual studies | 14 | To facilitate the evaluation of risk of bias in every research study, we will apply the COSMIN criteria for systematic reviews of PROMs [19, 25]. The 116-item checklist of COSMIN’s Risk of Bias consists of ten criteria, as detailed in Table 4, to qualify each measurement property for risk of bias including validity (e.g., content, structural, and criterion validity), reliability (e.g., stability and measurement error), responsiveness, and interpretability. The assessment will be based on a four-point rating scale: “very good”, “adequate”, “doubtful”, and “inadequate”. The quality of measurement properties will be graded as sufficient (+), insufficient (-), or indeterminate (?) based on the COSMIN criteria for good measurement properties. |
| Data synthesis | 15a | Prior to data synthesis and if feasible, data pooling will be conducted. A standard psychometric meta-analysis approach, posited by Hunter and Schmidt [28, 29] will be performed based on the psychometrics principles. This approach suggests that measurement errors (caused by unreliable measures), errors in sampling process, and range limitations are some of the sources that cause artifact variability and account for a large portion of the observed variation in the relationship between two variables in original studies. Consequently, it is crucial to conduct meta-analyses to identify potential moderating factors influencing these relationships and address artifact variability across studies. This can be achieved through the choice of study design or by subtracting it from the overall observed variability. |
|  | 15b | Meta-analysis will be on the basis of Fisher’s Z transformed partial correlation coefficient, known to have the lowest root mean square error and bias [30] in comparison with the partial correlation coefficients of meta-analysis [31]. The standardized effect size will be the Fisher’s Z that ranges from −∞ to +∞ and the standards used to interpret them are similar to those used for a correlation coefficient. If intraclass, Pearson, or Spearman correlations are provided, we will apply the Fisher’s variance stabilizing transformation [32, 33] to convert them into Fisher’s Z scores. If the coefficients of unstandardized beta and *F*-ratios were provided, we will primarily convert them to *r* and afterwards to Fisher’s Z score [32, 33]. If only *p* values are given, we will convert them to Z-score, *r*, and Fisher’s Z, respectively [33]. We will extract the overall effect size for each psychometric property and the effect sizes for each follow-up interval from studies that include follow-up assessments.  Data analysis will be carried out utilizing Comprehensive Meta-Analysis v.3 software [34]. With the presumption that heterogeneity is probable and that the mean of effect size is not stable across studies, random-effects models will be applied. To assess heterogeneity, the Cochrane’s Q test (the presence of heterogeneity) and the *I*^2^ statistic (diversity in heterogeneity effect estimates) will be evaluated [35]. Based on the standard interpretation [36], *I*^2^ statistic will be deemed as “not important” (0–40%), “moderate” (30–60%), “substantial” (50–90%), and “considerable” (75–100%). |
|  | 15c | Furthermore, where appropriate, funnel plots will be provided to identify reporting bias and the effects of small studies [36]. To ensure that the meta-analysis’s findings are robust in case of considerable heterogeneity, a sensitivity analysis will be conducted. |
|  | 15d | We will create a qualitative abstract based on the studies’ outcomes concerning the measurement properties of each tool when it is not feasible to pool the data. If there are discrepancies between the findings of various studies, possible explanations will be provided. If a consistent pattern appears, we will consolidate the results for each subgroup with consistent findings. In case no clear justification or discernible pattern emerges, the majority of the findings will be employed to assess the results. |
| Meta-bias(es) | 16 | As described in Table 5, the quality of result of every study will be evaluated via the COSMIN criteria for good measurement properties. These properties will be rated as “insufficient, (-)” “indeterminate, (?)” or “sufficient (+)”. The total score of a property will be equal to the lowest score it obtains, and its interpretation will be on the basis of the COSMIN criteria: 50% (high quality), 30–50% (moderate quality), and less than 30% (low quality) [19, 37, 38]. Four domains of reliability, validity, responsiveness, and interpretability will be included in the quality assessment taxonomy. This study will assess the quality of Exploratory Factor Analysis (EFA) using the guidelines outlined by Terwee, Bot [39]. According to these guidelines, in the absence of a theoretical or empirically emerged structural model, EFA is preferable. In contrast, when a model has already been theoretically proposed and/or has empirically emerged in the literature, should Confirmatory Factor Analysis (CFA) be tested [40, 41]. The results of EFA’s quality assessment will be interpreted as follows: The chosen factors can explain at least 50% of the variance OR they can explain less than 50% of the variance but a justification for this selection is proposed by authors (+);the vague or incomplete information (e.g., failure to mention the explained variance) prevents scoring the EFA’s quality (?); and criteria for a “plus” rating was not met (-) [39]. |
| Confidence in cumulative evidence | 17 | The Grading of Recommendations, Assessment, development, and evaluation (GRADE) working group approach [43] will be used to test the credibility of each piece of research posterior to providing a summary of general ratings on each psychometric property. The quality of evidence will be examined in five categories of risk of bias, publication bias, imprecision, inconsistency, and indirectness. Two researchers (PSY and MHA) independently appraise the overall quality of summarized findings. In the event of any disagreement, a third researcher (ETC) will decide. The quality of outcomes will be determined to be high (indicating a high degree of confidence in the measurement property’s estimate being close to the true value ), moderate (indicating a reasonable belief that the true estimate of the measurement property is likely close to the estimated value), low (suggesting a substantial potential for a significant difference between the true estimate and the estimated property), or very low (indicating a high likelihood of a substantial deviation between the actual measurement property and its estimated value). |

*** It is strongly recommended that this checklist be read in conjunction with the PRISMA-P Explanation and Elaboration (cite when available) for important clarification on the items. Amendments to a review protocol should be tracked and dated. The copyright for PRISMA-P (including checklist) is held by the PRISMA-P Group and is distributed under a Creative Commons Attribution Licence 4.0.**

*From: Shamseer L, Moher D, Clarke M, Ghersi D, Liberati A, Petticrew M, Shekelle P, Stewart L, PRISMA-P Group. Preferred reporting items for systematic review and meta-analysis protocols (PRISMA-P) 2015: elaboration and explanation. BMJ. 2015 Jan 2;349(jan02 1):g7647.*
