# Supplementary material for: Systematic review and meta-analysis of developmental assets scales: A study protocol for psychometric properties
Source: PLoS One. 2024 Sep 10;19(9):e0309909. doi: 10.1371/journal.pone.0309909 (PMC11386475; doi:10.1371/journal.pone.0309909)
Supplement: Attachment — Submitted filename: Reviewer Comment Plos One.docx [file pone.0309909.s002.docx]

Thanks for receiving the opportunity to review your paper. Overall, I appreciate the thoroughness and importance of your study in assessing the psychometric properties of developmental assets scales. Your focus on systematically reviewing and conducting a meta-analysis adds significant value to the existing literature. However, the manuscript must undergo minor revisions suggested below before it can be considered for publication.

I suggest a minor adjustment in the structure of the Abstract. I recommend you to consider incorporating your key results and conclusions section in place of the discussion section.

The introduction provides comprehensive background information that is highly relevant to the study. However, the authors must significantly expand on it by highlighting the significance and novelty of the study.

In methodology, address how you will assess and manage heterogeneity among the included studies. Mention all the statistical tests used in the study.

I noted an issue with the PRISMA flowchart presented as Figure [1]. It appears that the chart is incomplete as the value of 'k' (number of studies included in the meta-analysis) is not reflecting accurately. This discrepancy undermines the clarity and transparency of your methodology, which are essential aspects of systematic reviews and meta-analyses. I kindly request that you review and revise the PRISMA flowchart to ensure that it accurately represents the number of studies included in your analysis. Providing an accurate depiction of the study selection process is crucial for readers to assess the rigor and comprehensiveness of your review.

In result section, the data are well-organized. However, I suggest to add a well-constructed forest plot which can help to concisely convey the results of a meta-analysis. Incorporating this graphical depiction will not only aid in understanding the individual study contributions but also provide a holistic view of the overall effect size and its variability. Ensure consistency in the representation of odds ratios across the forest plot.

In discussion section, it should be more objective and clearer according to the purpose of the study and focus more on potential implications of your study's findings for both research and practice. Also add future research direction based on current findings.

Expand the reference list as systematic reviews and meta-analyses are expected to include a comprehensive collection of literature.

I suggest English language revisions and other typos and grammar errors should be corrected.
